# The Role of Ubiquitin-Proteasome Pathway and Autophagy-Lysosome Pathway in Cerebral Ischemia

**DOI:** 10.1155/2020/5457049

**Published:** 2020-01-30

**Authors:** Chunli Chen, Haiyun Qin, Jieqiong Tan, Zhiping Hu, Liuwang Zeng

**Affiliations:** ^1^Department of Neurology, Second Xiangya Hospital, Central South University, Changsha, Hunan 410011, China; ^2^Center for Medical Genetics, School of Life Sciences, Central South University, Changsha 410078, China; ^3^Hunan Key Laboratory of Medical Genetics, Central South University, Changsha 410078, China; ^4^Hunan Key Laboratory of Animal Model for Human Diseases, Central South University, Changsha 410078, China

## Abstract

The ubiquitin-proteasome pathway and autophagy-lysosome pathway are two major routes for clearance of aberrant cellular components to maintain protein homeostasis and normal cellular functions. Accumulating evidence shows that these two pathways are impaired during cerebral ischemia, which contributes to ischemic-induced neuronal necrosis and apoptosis. This review aims to critically discuss current knowledge and controversies on these two pathways in response to cerebral ischemic stress. We also discuss molecular mechanisms underlying the impairments of these protein degradation pathways and how such impairments lead to neuronal damage after cerebral ischemia. Further, we review the recent advance on the understanding of the involvement of these two pathways in the pathological process during many therapeutic approaches against cerebral ischemia. Despite recent advances, the exact role and molecular mechanisms of these two pathways following cerebral ischemia are complex and not completely understood, of which better understanding will provide avenues to develop novel therapeutic strategies for ischemic stroke.

## 1. Introduction

Protein homeostasis, the correct balance between production and degradation of proteins, is essential for cell function, development, and survival. Protein homeostasis also plays a critical role in cerebral ischemia. Protein homeostasis dysfunction has been implicated in the development and progression of neurological impairment in cerebral ischemia. Pharmacologic activation of the ATF6 arm of the unfolded protein response (UPR) reprograms cellular protein homeostasis and confers global neuroprotection in cerebral ischemia-reperfusion injury [1]. L-2-Oxothiazolidine-4-carboxylic acid (OTC), a cysteine precursor, improves proteostasis and protects against ischemic stroke injury [2].

The ubiquitin-proteasome pathway and autophagy-lysosome pathway are two major protein degradation systems in all eukaryotic cells to degrade a broad array of misfolded proteins. The ubiquitin-proteasome pathway is extremely efficient for the degradation of short-lived proteins and misfolded soluble proteins, while the autophagy-lysosome pathway is responsible for eliminating long-lived proteins, insoluble proteins, and certain whole organelles. The two highly regulated pathways do not only maintain protein homeostasis, but also mediate necrosis and apoptosis. In this review, we will focus on recent discoveries of the ubiquitin-proteasome pathway and autophagy-lysosome pathway and the crosstalk between them in the pathogenesis and treatment of cerebral ischemia and attempt to shed new light on the design of effective therapy strategies against cerebral ischemia injury-associated diseases.

## 2. The Ubiquitin-Proteasome Pathway in Cerebral Ischemia

### 2.1. Change of the Ubiquitin-Proteasome Pathway in Cerebral Ischemia

#### 2.1.1. Change of Ubiquitin in Cerebral Ischemia

The expression level of ubiquitin conjugates is greatly increased in plaques, especially unstable plaques, indicating that the ubiquitin-proteasome system is involved in the development of atherosclerotic plaques in intracranial arteries [3]. After hypoxia-ischemia, ubiquitinated proteins accumulate and proteasome biology in white matter of neonatal piglets is compromised [4]. The expression of ubiquitin increases in the peri-ischemic area after transient middle cerebral artery occlusion, with a peak at 72 hours and returning to the baseline levels until 7 days [5].

After transient cerebral ischemia, ubiquitin-conjugated proteins accumulate in Triton-insoluble aggregates. 763 peptides to 272 proteins, including proteins involved in important neuronal functions and signaling pathways, were highly enriched in these aggregates [6]. After middle cerebral artery occlusion, the formation of ubiquitin aggregates is driven by reperfusion rather than ischemia. Ubiquitin aggregates formed in potentially viable brain tissue may be later recruited into infarction by factors independent of ubiquitination [7].

#### 2.1.2. Change of Immunoproteasome in Cerebral Ischemia

Immunoproteasome, a subtype of proteasome, contains three major catalytic subunits: *β*1i, *β*2i, and *β*5i. The expression of the immunoproteasomal subunits, 20S *β*1i and *β*5i, is increased remarkably in the parietal cortex and hippocampus after transient cerebral ischemia, which is associated with a great increase in ubiquitinated proteins. The immunoproteasome induction after cerebral ischemia is supposed to play a critical role in coping with the damaged proteins and thus may be of great importance in neuronal survival and death [8]. The plasma level of the immunoproteasome is supposed to be a useful indicator in the early prediction of hemorrhagic transformation after acute ischemic stroke [9].

Immunoproteasome inhibition reduces infarction volume and attenuates inflammatory reaction after cerebral ischemia in rats, suggesting that selective immunoproteasome inhibitors may be promising candidates for the treatment of ischemic stroke [10]. In a rat model of focal cerebral ischemia, immunoproteasome inhibition also promotes angiogenesis, which is through enhancing hypoxia-inducible factor-1*α* (HIF-1*α*) abundance [11]. PR-957, a specific inhibitor of the immunoproteasome subunit low molecular weight polypeptide 7 (LMP7), confers neuroprotection in a mouse model of ischemic stroke, which is via modulating cytokine production and inhibiting Th17 differentiation [12].

### 2.2. The Role of the Ubiquitin-Proteasome Pathway in Cerebral Ischemia

After transient cerebral ischemia, the depletion of ubiquitin and the accumulation of its mutant form (ubiquitin(+1)) may inhibit the ubiquitin-proteasome pathway, which results in delayed neuronal death of CA1 pyramidal neurons directly or indirectly [13]. Meanwhile, proteasome activity may play a role in contralateral cortical plasticity occurring after focal cerebral ischemia [14]. Proteasome subunit type 6 (PSMA6) gene polymorphisms are associated with susceptibility to ischemic stroke, while PSMA6 (-C8G) gene polymorphism may play a protective role with the susceptibility of ischemic stroke [15]. The expression of key players of the ubiquitin-proteasome pathway (MuRF1 (muscle RING finger-1), MAFbx (muscle atrophy F-box), Musa1 (muscle ubiquitin ligase of SCF complex in atrophy-1)) is increased in both paretic and nonparetic skeletal muscles of patients with ischemic stroke and is critically associated with skeletal muscle atrophy after cerebral ischemia [16].

### 2.3. The Ubiquitin-Proteasome Pathway and the Treatment of Cerebral Ischemia

#### 2.3.1. The Ubiquitin-Proteasome Pathway Activation and the Treatment of Cerebral Ischemia

**Figure 1:**
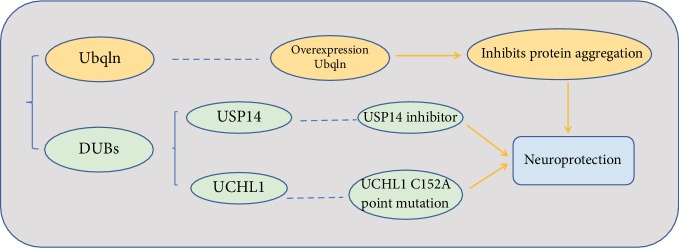
The ubiquitin-proteasome pathway activation strategy in the treatment of cerebral ischemia.

Protein aggregation has been proven to be a pathological basis responsible for ischemia-induced neuronal death. Therefore, enhanced removal of damaged proteins is supposed to be a promising therapeutic strategy for the treatment of ischemia-caused neuronal injury. Ubqln, a ubiquitin-like protein, mediates degradation of damaged proteins. Ubqln overexpression greatly inhibited the accumulation of protein aggregates and conferred neuroprotection against ischemia-induced cerebral injury [17]. Overexpression of Ubqln also protected mice from ischemic stroke and oxidative stress-caused neuronal injury, which was mediated by facilitating removal of misfolded proteins [18] (Figure 1).

Deubiquitinating enzymes (DUBs) are a large group of proteases that negatively regulates proteasome activity. The ubiquitin-specific protease 14 (USP14), one of the DUBs, is associated with the proteasome and regulates protein degradation. The USP14 inhibitor ameliorates neuronal injury and confers neuroprotection after cerebral ischemia-reperfusion insult in mice [19]. Ubiquitin C-terminal hydrolase L1 (UCHL1) is a unique brain-specific deubiquitinating enzyme. UCHL1 activity is of great importance for function after ischemic stroke, and the C152 site of UCHL1 plays a critical role in functional recovery after cerebral ischemia [20]. Cyclopentenone prostaglandins (CyPGs) are induced in the brain after ischemic stroke and disrupt the ubiquitin-proteasome system. The point mutation UCHL1 C152A protects primary neurons against cyclopentenone prostaglandin-induced cytotoxicity, indicating that it may also confer neuroprotection for postischemic neuronal injury. The neuroprotective effect was associated with less UCHL1 aggregation, as well as significantly less ubiquitinated protein accumulation and aggregation [21] (Figure 1).

Proteasome dysfunction leads protein aggregation after cerebral ischemia. Trehalose inhibits transient cerebral ischemia-induced protein aggregation via preservation of proteasome activity [22]. Propofol promotes PTEN degradation after cerebral ischemia injury via activating the ubiquitin-proteasome system and then attenuates hippocampal neuronal loss and memory impairment [23]. NSA administration inhibits the expression of mixed lineage kinase domain-like (MLKL) through the ubiquitination proteasome pathway and then greatly reduces infarct volume and improves neurological recovery after cerebral ischemia-reperfusion [24].

MicroRNA-124 confers neuroprotection in focal cerebral ischemia through inhibiting the deubiquitinating enzyme USP14 expression and reducing the expression level of REST [25]. Parkin promotes Drp1 degradation and exerts neuroprotective effect in oxygen-glucose deprivation/reperfusion injury [26]. However, the ischemia-induced ubiquitin E3 ligase tumor necrosis factor receptor-associated factor 6 (TRAF6) exacerbates cerebral ischemia injury through ubiquitinating and activating Rac1 [27] (Table 1).

Ischemic preconditioning protects against transient cerebral ischemic-induced damage through inhibiting ubiquitin aggregation [28]. Ischemic postconditioning also increases the activities of proteasomes and rescues focal ischemia-reperfusion-induced cerebral injury [29]. Meanwhile, elevated proteasome activity might contribute to the neuroprotective effect of glutamate preconditioning against cerebral ischemia-reperfusion-induced damage [30]. Excitotoxic stimulation with glutamate has multiple effects on the ubiquitin-proteasome system, which may account for the demise process in cerebral ischemia [31].

#### 2.3.2. The Ubiquitin-Proteasome Pathway Inhibition and the Treatment of Cerebral Ischemia

Hypoxia-inducible factor-1 (HIF-1) is a key regulator of cellular responses to hypoxia and can determine the fate of neurons during cerebral ischemia. HIF-1*α* degradation in ischemic neurons is mediated by 20S proteasomes. Proteasomal inhibition confers neuroprotection against cerebral ischemia through HIF-1*α* stabilization [32]. The novel proteasome inhibitor BSc2118 enhances angioneurogenesis and protects against cerebral ischemia-induced damage, which is via HIF-1*α* accumulation [33]. Preservation of blood-brain barrier integrity and reversal of peripheral immunosuppression also contributes to the neuroprotective effect of proteasome inhibitor BSc2118 against cerebral ischemia [34].

Early 2-methoxyestradiol (2ME2) administration inhibits neuronal HIF-1*α* through ubiquitin-proteasome system-mediated degradation [35]. Dexmedetomidine administration upregulates HIF-1*α* expression, reduces neuronal autophagy, and thus protects neurons against ischemia-reperfusion-induced damage [36].

Cellular prion protein protects neurons against ischemic-induced damage, promotes angioneurogenesis, and enhances neural progenitor cell homing through inhibiting proteasome activity [37]. Ginsenoside Rd confers neuroprotection and alleviates neurological deficits in ischemic stroke, and this neuroprotective effect is attributed to its capability of inhibiting microglial proteasome activity and sequential inflammation [38]. Ginsenoside Rg1 also attenuates ubiquitinated protein aggregation, suppresses inflammatory responses, and thus protects against cerebral ischemia-reperfusion-induced injury [39].

rAAV8-733-mediated gene transfer of CHIP/Stub-1 reduces the expression of ubiquitinated proteins, which contributes to the prevention of hippocampal neuronal death in an animal model of brain ischemia [40]. The *γ*-secretase blocker DAPT reduces blood-brain barrier (BBB) permeability during permanent brain ischemia, which is the result from decreased ubiquitination and degradation of occludin [41]. The nuclear factor erythroid 2-related factor 2 (Nrf2) pathway is a promising target for ischemic stroke therapy. The herb-derived compound, Britanin, selectively binds to a conserved cysteine residue, cysteine 151, of Keap1 and reduces Keap1-mediated ubiquitination of Nrf2, which results in activation of the Nrf2 pathway and attenuating cerebral ischemia-reperfusion-induced damage [42] (Table 2).

Proteasome inhibition preconditioning promotes ER stress and autophagy but inhibits inflammation and apoptosis. Therefore, the combination of an autophagy promoter and a proteasome inhibitor is supposed to be a new potential strategy for the therapy of disorders related to hypoxia-reperfusion or ischemia-reperfusion injury [43]. Meanwhile, ischemic postconditioning confers neuroprotection against cerebral ischemic insult through Hsp70-mediated proteasome inhibition and promotes neural progenitor cell transplantation [44].

### 2.4. Small Ubiquitin-Like Modifier (SUMO) in Cerebral Ischemia

#### 2.4.1. Change of SUMO in Cerebral Ischemia

The small ubiquitin-like modifier (SUMO), a ubiquitin-like protein, mediates posttranslational protein modifications and is involved in the regulation of a myriad of stress responses. Cerebral ischemia-reperfusion increases the aggregation of SUMO, which plays an important role in the altered protein dynamics after brain ischemia [45]. Focal cerebral ischemia also induces the expression of SUMO-conjugated proteins. After middle cerebral artery occlusion, SUMO2/3 is associated with ubiquitinated protein aggregates in the mouse neocortex [46]. Maintenance of a globally decreased expression of SUMO2/3 conjugation is a component of ischemic tolerance [47].

#### 2.4.2. The Role of SUMO in Cerebral Ischemia

Neuron-specific knockdown of SUMO inhibits global gene expression response and exacerbates the functional outcome in a mouse model of transient forebrain ischemia [48]. SUMOylation enhances neural stem cell graft survival and integration in ischemic stroke. Overexpression of the SUMO E2-conjugase Ubc9 in neural stem cells promotes neuronal differentiation and enhances resistance to cerebral ischemia-reperfusion-induced damage [49]. Ubc9 is also accounted for isoflurane preconditioning-induced neuroprotection in cerebral ischemic injury [50].

SUMO-specific protease 1 (SENP1) deconjugates SUMO from modified proteins and confers neuroprotection in cerebral ischemia. SENP1 inhibits SUMO1 conjugation and thus protects against ischemia-reperfusion-induced apoptosis [51]. URB597 inhibits SUMO-specific protease 3 (SENP3) and attenuates chronic cerebral hypoperfusion-induced neurovascular unit dysfunction in rats [52].

SUMOylation of NaV1.2 channels contributes to the early response to acute hypoxia in central neurons [53]. Ischemic preconditioning confers neuroprotection in ischemic stroke, which is mediated by SUMO1-induced SUMOylation of LYS590 of NCX3 f-Loop [54] (Table 3). E2-25K is an E2-conjugating enzyme and is SUMOylated during oxidative stress. E2-25K inhibits proteasome activity through its SUMOylation and thus plays a critical role in cerebral ischemia-reperfusion insult [55] (Table 3).

#### 2.4.3. SUMO and Hypothermia in Cerebral Ischemia

Hypothermia confers early neuroprotective effects after middle cerebral artery occlusion in rats, which is involved in the protein conjugation of SUMO2/3 [56]. Hypothermia also increases cellular tolerance to cerebral ischemia, which substantially results from increases in global SUMOylation [57]. Moderate hypothermia promotes bone marrow stromal cell resistance to hypoxia through enhancing protein SUMOylation. Therefore, bone marrow stromal cell transplantation along with moderate hypothermia is supposed to be a promising candidate for the treatment of ischemic stroke [58].

## 3. The Autophagy-Lysosome Pathway in Cerebral Ischemia

### 3.1. Change of the Autophagy-Lysosome Pathway in Cerebral Ischemia

**Figure 2:**
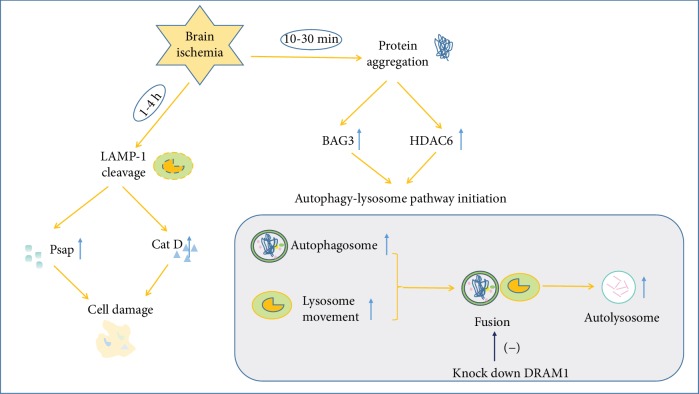
Change of the autophagy-lysosome pathway after brain ischemia.

When the ubiquitin-proteasome pathway is overloaded, excessive misfolded proteins will be removed by the autophagy-lysosome pathway. Depending on the BAG1/BAG3 ratio and expression level of HDAC6, a switch from the ubiquitin-proteasome pathway to the autophagy-lysosome pathway occurs between 10 and 30 min during cerebral ischemia [59]. The lysosomal enzymes are spilled into the cytoplasm 1-4 h after ischemia-reperfusion, indicating the rapid loss of lysosomal membrane integrity after ischemic stroke [60] (Figure 2). The dynamics of lysosomal movements is revealed by the phagocytic state of brain myeloid cells in the ischemic brain. Lysosomal clustering is proximal to the cell membrane initially and will be perinuclear finally, consistent with the initial stage of active target internalization and the final stage of phagocytosis or autophagy [61].

Lysosomal Psap processing is found to be changed after cerebral ischemia from proteomic analysis of synaptosomal protein expression. Cerebral ischemia adversely affects the synapse, which may be involved in regulating the neuronal viability after ischemic stroke [62]. The distribution of lysosomal proteins is also changed after hypoxia-ischemia in the rat hippocampus and cortex. The lysosomal proteins Psap and Cat D are released abnormally to the cytosol after hypoxia-ischemia injury, which is caused by LAMP-1 cleavage, and will result in cell damage [63] (Figure 2). Chronic brain hypoperfusion leads to cognitive decline and abnormal cellular protein accumulation, accompanied by the abnormal autophagic-lysosomal system. Reduced expression of LAMP-2 protein by mir-27a leads to inefficient lysosomal clearance after chronic brain hypoperfusion in rats [64].

Cerebral ischemia significantly increases autophagosomes and autolysosomes in hippocampal CA1 and DG neurons. Transient cerebral ischemia-induced protein aggregation on subcellular organelle membranes could result in multiple organelle damage, accompanied by delayed neuronal death [65]. DNA damage-regulated autophagy modulator protein 1 (DRAM1) is a multipass membrane lysosomal protein and is involved in autophagy. Cerebral ischemia-reperfusion injury induces the expression of DRAM1 protein and leads to autophagy activation. Knockdown of DRAM1 blocks autophagosome-lysosome fusion, inhibits autophagy, and aggravates cerebral ischemia-induced cell damage [66] (Figure 2). The membrane-bound water channel aquaporin-4 (AQP4) is of great importance in maintaining water homeostasis in the brain. Ischemia induces AQP4 internalization in the brain, and the internalized AQP4 will be degraded by the lysosome [67].

### 3.2. The Role of the Autophagy-Lysosome Pathway in Cerebral Ischemia

#### 3.2.1. The Neuroprotective Role of the Autophagy-Lysosome Pathway in Cerebral Ischemia

Absence of the circadian clock protein PER1 inhibits the hippocampal autophagic machinery and contributes to vulnerability to cerebral ischemia injury [68]. Neonatal hypoxic-ischemia initially activates autophagy but later impairs autophagosome clearance and leads to neuronal death in a piglet model [69].

**Figure 3:**
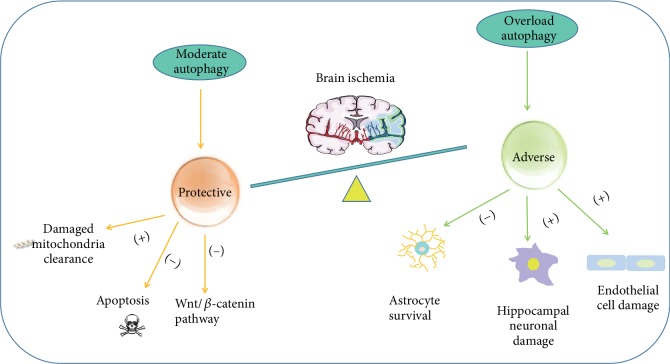
Dual roles of the autophagy-lysosome pathway in brain ischemia.

Autophagy plays diverse roles in cerebral ischemia-reperfusion injury (Figure 3). Mitophagy-related mitochondrial clearance and downstream apoptosis inhibition account for the neuroprotective effect of autophagy in cerebral ischemia-reperfusion injury. PARK2 may participate in the mitophagy process responsible for the protective role of autophagy [70]. Autophagy negatively regulates the Wnt/*β*-catenin pathway and thus exerts protective effect in neural ischemia and hypoxia [71].

#### 3.2.2. The Adverse Role of the Autophagy-Lysosome Pathway in Cerebral Ischemia

The autophagic-lysosomal pathway is activated in injured astrocytes following glucose and oxygen deprivation and focal cerebral ischemia, which at least partly account for decreased survival of astrocytes [72]. Asphyxia cardiac arrest-induced cerebral ischemia-reperfusion enhances the expression of lysosomal proteins and autophagosome numbers in hippocampal cells, as well as leads to hippocampal neuronal damage, which is attenuated by autophagy inhibition and lysosome inhibition [73].

RIP1K activates the autophagic-lysosomal pathway and induces necroptosis, which may account for the neuronal and the astrocytic cell death after cerebral ischemia [74]. The endothelial cell is damaged after sustained ischemic injury, and the autophagy-lysosome signaling is at least partially activated by peroxynitrite-mediated nitrosative stress [75] (Figure 3).

### 3.3. The Autophagy-Lysosome Pathway and the Treatment of Cerebral Ischemia

#### 3.3.1. The Autophagy-Lysosome Pathway Activation and the Treatment of Cerebral Ischemia

Ischemia-reperfusion induces autophagosome formation and inhibits autolysosome degradation. Mitofusin 2, a mitochondrial fusion protein, enhances autophagosome formation and promotes the fusion of autophagosomes and lysosomes and, thus, attenuates ischemia-reperfusion-induced damage [76]. Rab7b, a lysosome-associated small Rab GTPase, regulates autophagy during cerebral ischemia and confers neuroprotection against ischemic brain damage [77]. The autophagic flux is found to be impaired in an animal model of ischemic stroke. Pseudoginsenoside-F11 ameliorates ischemic brain damage through attenuating autophagic-lysosomal defects [78].

Sphingosine kinase 2 exerts a neuroprotective effect in cerebral ischemia. It could interact with Bcl-2 via its BH3 domain, thereby dissociating it from Beclin-1, and activating autophagy subsequently [79]. Phosphorylated CAV1 binds to the BECN1/VPS34 complex under oxidative stress, which thereby induces the activation of autophagy, which contributes to its neuroprotection effect in ischemic injury [80]. Neuronal Rho GTPase Rac1 ablation protects against ischemic brain damage through maintenance of lysosomes [81].

Resveratrol induces Sirt1-dependent autophagy, which thereby suppresses NLRP3 inflammasome activation, and thus protects against cerebral ischemia-reperfusion injury [82]. Hyperhomocysteinemia (HHcy) is a common risk factor for cerebral ischemia. HHcy inhibits lysosomal membrane protein expression, which thereby leads to lysosomal dysfunction and autophagic defect, which can be alleviated by vitamin B supplementation [83]. CysC is a critical determinant accounting for endogenous neuroprotection and is a promising agent for the therapy of stroke, which is mediated by preserving lysosomal membrane integrity [84]. Acute ethanol exposure alleviates acidosis-induced neurotoxicity after cerebral ischemia, which is mediated by increased acid-sensing ion channel 1a (ASIC1a) protein degradation through the autophagy-lysosome pathway [85] (Table 4).

Autophagosome accumulation is increased after oxygen-glucose deprivation insult, which is mediated by increased autophagosome formation and decreased autophagosome clearance. Sevoflurane postconditioning protects against oxygen-glucose deprivation injury through inhibition of autophagosome accumulation [86]. Cerebral ischemia-induced CA1 neuronal death is exacerbated after inhibition of autophagosome-lysosome fusion. Hypoxic preconditioning protects neurons against transient global cerebral ischemia injury through Rab7-mediated autophagosome maturation [87].

Mild hypothermia confers protection against hippocampal neuronal injury after oxygen-glucose deprivation/reperfusion, which is mediated by promoting lysosomal function and autophagic flux [88]. Remote ischemic preconditioning protects against cerebral ischemia-reperfusion injury through activation of the autophagy-lysosome pathway [89].

#### 3.3.2. The Autophagy-Lysosome Pathway Inhibition and the Treatment of Cerebral Ischemia

miR-207 and miR-352 play important roles in regulating cerebral ischemic damage and spontaneous recovery through the lysosomal pathway. miR-207 mimics could decrease the expression of cellular lysosome and autophagosome and, thus, induce the expression of autophagic vacuoles [90]. Dapsone (DDS), an anti-inflammation and antioxidation drug, suppresses abnormal degradation of tight junction ZO-1 through autophagy and inhibits lysosome accumulation and, thus, confers neuroprotection on cerebral microvessels [91].

2-(3′,5′-Dimethoxybenzylidene) cyclopentanone (DMBC), a novel synthetic small-molecule compound, inhibits the release of cathepsin B from the lysosomes into the cytoplasm after cerebral ischemia, thereby protecting neurons against ischemic injury [92]. CA-074Me or Clik148, a selective inhibitor of cathepsin B or cathepsin L, inhibits the release of cathepsin B or L from the lysosomes into the cytoplasm and activation of caspase-3 in astrocytes after the permanent middle cerebral artery occlusion (pMCAO). Suppression of cysteine cathepsin B and L activation could inhibit the tBid-mitochondrial apoptotic signaling pathway, thereby protecting astrocytes against ischemic injury [93]. 3-MA and Wort inhibit autophagy and protect astrocytes against ischemic injury. Inhibition of autophagy induces the expression of the lysosomal Hsp70.1B in ischemic astrocytes, which thereby stabilizes lysosomal membranes, and subsequently blocks the cathepsin-tBid-mitochondrial apoptotic signaling pathway [94]. Besides cathepsin B inhibition, CA074-me may exert the neuroprotective effect by preserving lysosomal membrane integrity and suppressing lysosomal rupture [95].

Fingolimod inhibits ischemia-induced neuronal autophagy via the mTOR/p70S6K signaling pathway and confers neuroprotection against cerebral ischemic insult [96]. *Lycium barbarum* polysaccharide protects against ischemia-reperfusion injury in primary cultured hippocampal neurons, which is mediated by inhibition of apoptosis and autophagic cell death through the PI3K/Akt/mTOR pathway [97]. Cornin also suppresses autophagy in SH-SY5Y cells after oxygen and glucose deprivation, which is via regulation of the PI3K/Akt/mTOR signaling pathway [98] (Table 5).

Hyperbaric oxygen (HBO) suppresses autophagy activity and inhibits apoptosis and necrosis levels, thereby conferring neuroprotection against ischemic brain injury [99].

Sevoflurane postconditioning suppresses the activation and release of lysosomal cathepsin B and alleviates reactive astrogliosis and glial scar formation after cerebral ischemia-reperfusion [100].

## 4. The Crosstalk between the Ubiquitin-Proteasome Pathway and the Autophagy-Lysosome Pathway in Cerebral Ischemia

Ubiquitylation is a common component that guides misfolded protein to the proper degradation system for both the ubiquitin-proteasome pathway and the autophagy-lysosome pathway [101]. Besides, the two pathways recognize their substrates through corresponding ubiquitin tags. K48-linked ubiquitin chains are predominantly introduced to be a signal for the ubiquitin-proteasome pathway, while K63-linked ubiquitin chains are known as a signal for autophagic degradation [102, 103]. Despite their mode of function and that their ubiquitin tags for substrate recognition are different, there is crosstalk between the ubiquitin-proteasome pathway and the autophagy-lysosome pathway in the pathogenesis of cerebral ischemia.

On the one hand, initial evidences about functional links between the ubiquitin-proteasome pathway and the autophagy-lysosome pathway reveal that inhibition of one can result in a compensatory upregulation of the other pathway [104]. This can be observed in in vivo and in vitro models of cerebral ischemia. Liu et al. demonstrate that the high level of the HDAC6 and BAG1/BAG3 ratio decides the switch from the proteasome pathway to autophagy between 10 min and 30 min of cerebral ischemia [59]. Besides, inhibition of the ubiquitin-proteasome pathway by MG132 could activate autophagy and induce aggresome formation in primary neurons [105]. In addition, Quiroga et al. propose that Herp (an ER stress-induced stress protein) plays a key role in mediating the proteasomal degradation of autophagy regulator Beclin-1 through the regulation of the E3 ubiquitin ligase Hrd1. They further demonstrate that in a cell glucose starvation model, depletion or possibly inhibition of Herp leads to decreased Beclin-1 proteasomal degradation, which triggers a compensatory autophagy upregulation [106].

On the other hand, mitophagy is also a prominent biological phenomenon that involves both the ubiquitin-proteasome pathway and the autophagy-lysosome pathway since the ubiquitin-proteasome activity has been proven to be a prerequisite in the preparation of selective autophagy for mitochondria [107]. This scenario is also observed in cerebral ischemia. Lan et al. demonstrate that severe cellular stress following cerebral ischemia can cause significant aggregation of PINK1 in the mitochondrial outer membrane and subsequent Parkin phosphorylation, which in turn ubiquitylates mitochondrial targets as well as promotes the autophagic degradation of mitochondria [106].

## 5. Conclusions and Future Directions

Collectively, this review provides novel insights into the role of the ubiquitin-proteasome pathway and the autophagy-lysosome pathway after cerebral ischemia. Cerebral ischemia leads to dysfunction of these two pathways, which contributes to misfolded protein accumulation and neuronal necrosis and apoptosis ultimately. Regulation of these two pathways may be an important mechanism for the neuroprotective effects of many therapeutic agents and approaches against cerebral ischemia.

The challenge in the future research is to determine the key factors that mediate degradation of apoptosis and oxidative damage-related proteins, as well as the specific receptors that mediate the degradation of mitochondria, the endoplasmic reticulum, and Golgi apparatus through the autophagy-lysosome pathway in cerebral ischemic injury. To achieve this, mass spectrometry can be employed to quantitatively analyze proteins that are altered on organelles during cerebral ischemia, especially those whose expression is increased.

At the same time, future researches will also need to clarify the dynamic changes of the ubiquitin-proteasome pathway and the autophagy-lysosome pathway at different time points during cerebral ischemia, which will help us to determine their specific role in cerebral ischemic injury. As we know, there are transgenic mice expressing Ub^G76V^-GFP, an ubiquitin-proteasome system reporter, for monitoring the role of ubiquitin-proteasome-dependent proteolysis [108], transgenic mice systemically expressing RFP-EGFP-LC3 for real-time observation of autophagy [109] and a mito-QC reporter mouse model for mammalian mitophagy detection [110], which will help us to solve these issues. These future studies will discover new measures targeting these two pathways to maintain protein homeostasis and provide new avenues to treat cerebral ischemic-related diseases.

## Figures and Tables

**Table 1:** Agents that can activate proteasome activity in ischemic stroke models.

Agents	Effects on ischemic stroke	Possible mechanisms	Models	References
Trehalose	Protective	Preservation of proteasome activity	In vitro and in vivo	[22]
Propofol	Protective	Promoting PTEN degradation	In vivo	[23]
NSA	Protective	Promoting MLKL degradation	In vivo	[24]
MicroRNA-124	Protective	Inhibiting the deubiquitinating enzyme USP14 expression and reducing the expression of REST	In vitro and in vivo	[25]
Parkin	Protective	Promotes Drp1 degradation	In vitro	[26]
TRAF6	Detrimental	Ubiquitinating and activating Rac1	In vivo	[27]

Abbreviations: PTEN: phosphatase and tensin homology deleted on chromosome ten; USP14: ubiquitin-specific protease 14; MLKL: mixed lineage kinase domain-like; REST: RE1-silencing transcription factor; Drp1: dynamin-related protein 1; TRAF6: tumor necrosis factor receptor-associated factor 6.

**Table 2:** Agents that can inhibit proteasome activity in ischemic stroke models.

Agents	Effects on ischemic stroke	Possible mechanisms	Models	References
Cellular prion protein	Protective	Inhibiting proteasome activity, promoting angioneurogenesis, and enhancing neural progenitor cell homing	In vivo	[37]
Ginsenoside Rd	Protective	Inhibiting microglial proteasome activity and sequential inflammation	In vivo and in vitro	[38]
Ginsenoside Rg1	Protective	Attenuating ubiquitinated protein aggregation and inflammation	In vivo	[39]
rAAV8-733-mediated gene transfer of CHIP/Stub-1	Protective	Reducing the expression of ubiquitinated proteins	In vivo and in vitro	[40]
*γ*-secretase blocker DAPT	Protective	Decreasing ubiquitination and degradation of occludin	In vivo	[41]
Britanin	Protective	Reducing Keap1-mediated ubiquitination of Nrf2	In vivo and in vitro	[42]

Abbreviations: rAAV: recombinant adenoassociated virus; Nrf2: nuclear factor erythroid 2-related factor 2.

**Table 3:** The role of SUMOylation pathway in cerebral ischemia.

Molecular sites targeting SUMO	Possible mechanisms	Models	References
Neuron-specific knockdown of SUMO	Exacerbating functional outcome	In vivo	[48]
Overexpression the SUMO E2-conjugase Ubc9	Promoting neuronal differentiation and enhancing resistance	In vitro	[49]
SUMO-specific protease 1 (SENP1)	Inhibiting SUMO1 conjugation and conferring neuroprotection	In vivo	[51]
URB597	Inhibiting SENP3 and attenuating chronic cerebral hypoperfusion	In vivo	[52]
SUMOylation of LYS590 of NCX3 f-Loop	Conferring neuroprotection	In vivo	[54]
SUMOylation of E2-25K	Inhibiting proteasome activity	In vivo	[55]

**Table 4:** Agents that can activate the autophagy-lysosome pathway in ischemic stroke models.

Agents	Effects on ischemic stroke	Possible mechanisms	Models	References
Mitofusin	Protective	Enhancing autophagosome formation and promoting the fusion of autophagosomes	In vitro	[76]
Rab7b	Protective	Regulating the lysosomal degradation of TLR4	In vivo	[77]
PF-11	Protective	Attenuating autophagic-lysosomal defects	In vivo	[78]
Sphingosine kinase 2	Protective	Activating autophagy	In vitro	[79]
Phosphorylated CAV1	Protective	Activating autophagy	In vivo and in vitro	[80]
Neuronal rho GTPase Rac1 ablation	Protective	Maintenance of lysosomes	In vivo	[81]
Resveratrol	Protective	Suppressing NLRP3 inflammasome activation	In vivo	[82]
HHcy	Detrimental	Promoting lysosomal dysfunction and autophagic defect	In vivo and in vitro	[83]
CysC	Protective	Preserving lysosomal membrane integrity	In vivo	[84]
Acute ethanol exposure	Protective	Increased ASIC1a protein degradation	In vitro	[85]

Abbreviations: TLR4: toll-like receptor 4; PF-11: pseudoginsenoside-F11; CAV1: caveolin1; NLRP3: NOD-like receptor family pyrin domain-containing 3; HHcy: hyperhomocysteinemia; CysC: cystatin C; ASIC1a: acid-sensing ion channel 1a.

**Table 5:** Agents that can inhibit the autophagy-lysosome pathway in ischemic stroke models.

Agents	Effects on ischemic stroke	Possible mechanisms	Models	References
miR-207 mimics	Protective	Decreasing the expression of cellular lysosome and autophagosome	In vivo	[90]
DDS	Protective	Inhibiting lysosome accumulation	In vivo and in vitro	[91]
DMBC	Protective	Inhibiting the release of cathepsin B from the lysosomes into the cytoplasm	In vivo and in vitro	[92]
Clik148	Protective	Inhibiting the release of cathepsin L from the lysosomes into the cytoplasm and activation of caspase-3	In vivo and in vitro	[93]
3-MA and Wort	Protective	Inhibiting autophagy and stabilizing lysosomal membranes	In vivo and in vitro	[94]
CA074-me	Protective	Inhibiting the release of cathepsin B, preserving lysosomal membrane integrity, and suppressing lysosomal rupture	In vivo	[93, 95]
Fingolimod	Protective	Inhibiting autophagy via the mTOR/p70S6K pathway	In vivo	[96]
*Lycium barbarum* polysaccharide	Protective	Inhibiting apoptosis and autophagy through the PI3K/Akt/mTOR pathway	In vitro	[97]
Cornin	Protective	Inhibiting autophagy through the PI3K/Akt/mTOR pathway	In vitro	[98]

Abbreviations: DDS: dapsone; DMBC: 2-(3′,5′-dimethoxybenzylidene) cyclopentanone; 3-MA: 3-methyladenine; Wort: wortmannin.
